# The antioxidant effect of tetrahedral framework nucleic acid‐based delivery of small activating RNA targeting DJ‐1 on retinal oxidative stress injury

**DOI:** 10.1111/cpr.13635

**Published:** 2024-04-09

**Authors:** Qiaowei Wu, Jingyi Zhu, Xianggui Zhang, Xiaoxiao Xu, Delun Luo, Yunfeng Lin, Ming Yan, Yanping Song

**Affiliations:** ^1^ Department of Ophthalmology General Hospital of Central Theater Command Wuhan China; ^2^ Innovative Institute of Chinese Medicine and Pharmacy Chengdu University of Traditional Chinese Medicine Chengdu China; ^3^ Department of Maxillofacial Surgery, State Key Laboratory of Oral Diseases West China Hospital of Stomatology Chengdu China

## Abstract

Age‐related macular degeneration (AMD) and diabetic retinopathy (DR) are the world's leading causes of blindness. The retinal pigment epithelium (RPE) and vascular endothelial cell exposed to oxidative stress is the major cause of AMD and DR. DJ‐1, an important endogenous antioxidant, its overexpression is considered as a promising antioxidant treatment for AMD and DR. Here, we modified the tetrahedral frame nucleic acids (tFNAs) with DJ‐1 saRNAs as a delivery system, and synthesized a novel nanocomplex (tFNAs‐DJ‐1 saRNAs). In vitro studies show that tFNAs‐DJ‐1 saRNAs can efficiently transfer DJ‐1 saRNAs to human umbilical vein endothelial cells (HUVECs) and ARPE‐19s, and significantly increased their cellular DJ‐1 level. Reactive oxygen species expression in H_2_O_2_‐treated HUVECs and ARPE‐19s were decreased, cell viability was enhanced and cell apoptosis were inhibited when tFNAs‐DJ‐1 saRNAs were delivered. Moreover, tFNAs‐DJ‐1 saRNAs preserved mitochondrial structure and function under oxidative stress conditions. In the aspect of molecular mechanism, tFNAs‐DJ‐1 saRNAs activated Erk and Nrf2 pathway, which might contribute to its protective effects against oxidative stress damage. To conclude, this study shows the successfully establishment of a simple but effective delivery system of DJ‐1 saRNAs associated with antioxidant effects in AMD and DR, which may be a promising agent for future treatment in oxidative stress‐related retinal disorders.

## Graphical abstract

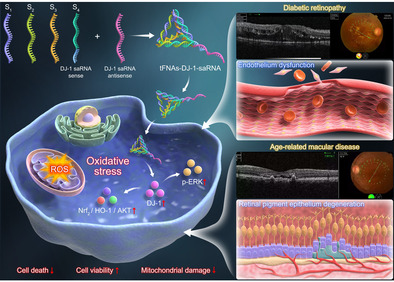

## 1 INTRODUCTION

Oxidative stress plays a pivotal role in developing and accelerating retinal disorders including age‐related macular degeneration (AMD) and diabetic retinopathy (DR).^1^, ^2^ Factors like aging, gene abnormalities and chronic exposure to hyperglycaemia bring about excessive reactive oxygen species (ROS) formation. An imbalance between ROS and antioxidant scavenger (in favour of pro‐oxidant species) in retinal pigment epithelium (RPE) and retinal endothelial cells leads to inflammation, mitochondrial dysfunction and cell degenerations, which in conjunction result in the damage of blood‐retinal barrier integrity and retinal neuronal dysfunction.^3^ Therefore, antioxidant therapies are considered promising treatment for AMD and DR.

Protein deglycase DJ‐1 is a redox‐sensitive protein encoded by Parkinson disease Protein 7 (PARK7) gene. As an important endogenous antioxidant, it rises upon oxidative stresses in many human diseases including retinal ischemia reperfusion injury, neurodegenerative diseases and diabetes mellitus.^4^, ^5^ DJ‐1 is found to protect cells against oxidative stress‐induced damage by regulating transcriptional signalling, ROS clearance, and mitochondrial function, and is deemed an optimal target for antioxidant therapies. Studies have confirmed that DJ‐1 overexpression can reduce high glucose‐induced oxidative stress and apoptosis of retinal pericytes through the PI3K/AKT/mTOR and Nrf2 signalling pathway, thereby preventing the progression of DR.^6^, ^7^ DJ‐1 is robustly expressed in RPE.^8^ Loss of DJ‐1 sabotaged the RPE antioxidant machinery in mice, resulting in RPE thinning and alterations in the electroretinograms.^9^ Therefore, increasing the DJ‐1 expression in RPE and endothelial cells serve as a very striking therapeutic method for DR and AMD management.

In view of the importance of DJ‐1 protein as an antioxidant, a lot of efforts have been made to enhance its expression in various diseases like Parkinson's disease.^10^ The strategies are mainly viral‐based vectors including adenoviruses and lentiviruses.^11^, ^12^ Although proven effective, some inherent drawbacks such as potential immunogenicity, cellular toxicity and the risk of insertional mutagenesis could not be ignored.^13^ Therefore, we facilized a new technique called small activating RNAs (saRNAs) to induce DJ‐1 expression. saRNAs are short double‐stranded RNAs that can selectively promote the transcription of a gene by targeting its promoter region.^14^ It presents a new pathway for RNA‐based gene upregulation, and was successfully utilized in cancer research.^15^ Despite its high potential, delivery challenges slow down its clinical application. RNA activation (RNAa) molecules are vulnerable to enzymatic digestion, fast cleared from the bloodstream, and have poor cellular uptake. All these characteristics calls for promising drug delivery systems to improve the pharmacokinetics of saRNAs. Delivery vehicles like lipid nanoparticles and aptamers have been tried for increasing in vivo saRNA transfer,^16^ and novel delivery technologies will be invaluable for RNAa development.

In recent years, tetrahedral framework nucleic acids (tFNAs) have emerged as a hot drug delivery carrier.^17^ tFNAs is a new class of three‐dimensional (3D) DNA nanomaterial, which are made of four single‐stranded DNAs (ssDNAs). tFNAs has many fine natures, such as simple synthesis, desirable cell‐entry performance, satisfying biocompatibility and high biosafety,^17^ which revealed itself as a potential vehicle for saRNAs. It has been successfully used in delivering antimicrobial peptides, angiogenic peptide and anticancer drugs.^18^, ^19^, ^20^ With the help of tFNAs, DJ‐1‐saRNA degradation could be effectively reduced, and cellular endocytosis and tissue permeability could be significantly enhanced. Moreover, tFNAs itself is deemed an antioxidant nanostructure, and has been found to exhibit remarkable antioxidant effects in diseases like acute kidney injury, diabetic wound healing and periodontitis,^21^, ^22^, ^23^ making it an ideal assistance to DJ‐1‐saRNA delivery.

**SCHEME 1:**
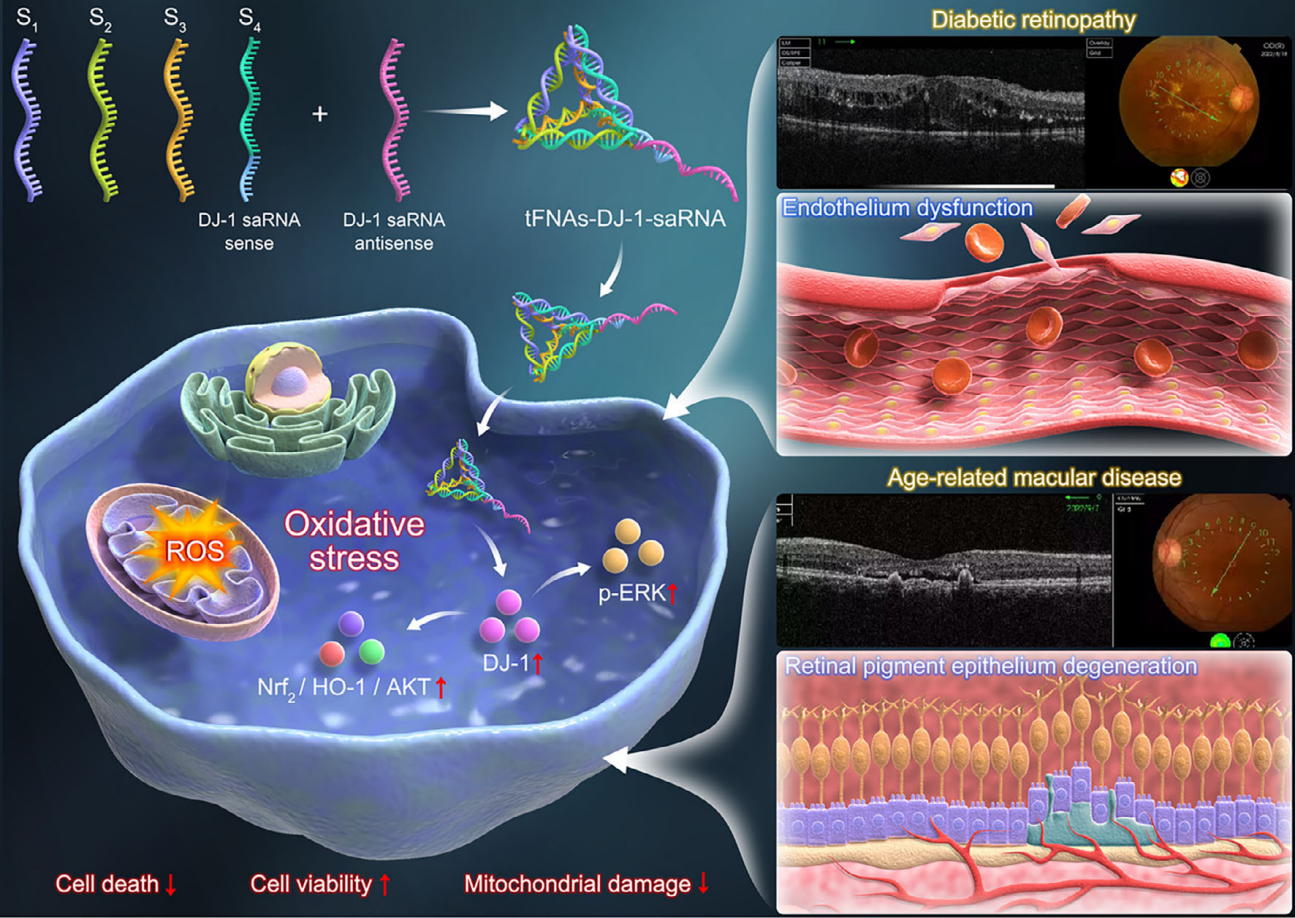
Illustration of the preparation of tFNAs‐DJ‐1 saRNAs complex and its anti‐oxidative effects on human umbilical vein endothelial cells (HUVECs) and ARPE‐19.

In this study, we successfully synthesized tFNA‐DJ‐1‐saRNA, then measured the characterization of tFNA‐DJ‐1‐saRNA and its cellular uptake. The combined tFNA and DJ‐1‐saRNA can significantly enhance the expression of DJ‐1 protein in RPEs and endothelial cells compared to DJ‐1‐saRNA solely. Then we conducted relevant in vitro studies, and confirmed that tFNA‐DJ‐1 saRNA showed a prominent protective effect on the RPEs and endothelial cells against oxidative stress through Erk and Nrf2 pathway. To the best of our knowledge, it is the first attempt to explore the combinative effects of tFNAs and saRNAs, and the initial application of tFNAs with DJ‐1‐saRNA confirms the great potential of this system in the treatment of oxidative stress‐related retinal diseases (Scheme 1).

## 2 METHOD

### 2.1 Synthesis and characterization of tFNAs‐DJ‐1‐saRNA

tFNAs‐DJ‐1‐saRNA was synthesized from three ssDNAs (S2, S3 and S4, Sangon Biotech, Shanghai, China, Table S1), one ssDNA modified by DJ‐1‐saRNA sense (S1‐saRNA sense, Table S1), and saRNA antisense (Table S1) according to the same synthesis method as tFNAs. Specifically, S1‐saRNA sense, saRNA antisense, S2, S3 and S4 were equally added to TM buffer (50 nM MgCl_2_, 10 nM Tris–HCl, pH = 8.0), blended well, heated to 75°C for 10 min, and cooled to 4°C for 30 min. The concentration of successfully synthesized tFNAs‐DJ‐1‐saRNA was 1 μM. Then, we used PAGE (8%, Biotime, Nanjing, China) to testify the successful synthesis of tFNAs and tFNAs‐DJ‐1‐saRNA based on their different mobility. High‐performance capillary electrophoresis (HPCE) was also used to reconfirm the successful synthesis of these nanomaterials. Moreover, the characterization of tFNA‐DJ‐1‐saRNA was investigated by transmission electron microscopy (TEM) and dynamic light scattering (DLS).

### 2.2 Cell culture and treatment

Human umbilical vein endothelial cells (HUVECs) and ARPE‐19 cells were bought from ATCC. Two cell lines were incubated in Dulbecco's modified Eagle Medium (DMEM basic, Gibco, USA) and DMEM Mixture F‐12 (DMEM/F‐12 basic, Gibco, USA), respectively, with 10% fetal bovine serum (FBS) and 1% streptomycin–penicillin in normoxia environment comprising 95% air and 5% CO_2_ at 37°C for 24 h. We prepared 62.5, 125 and 250 nM tFNAs‐DJ‐1‐saRNA solution by adding 1 μM tFNAs‐DJ‐1‐saRNA in volumes of 1/16, 1/8 and 1/4 of the total volume of the desired solution to DMEM and DMEM/F‐12, respectively. Next, HUVECs and ARPE‐19 cells were cultured in DMEM and DMEM/F‐12 containing different concentrations (100–800 μM) of H_2_O_2_ for 6 h, then cultured with DMEM and DMEM/F‐12 containing various concentrations (62.5, 125 and 250 nM) of DJ‐1‐saRNA, tFNAs, tFNAs‐DJ‐1‐saRNA for another 24 h.

### 2.3 Uptake of tFNAs‐DJ‐1‐saRNA by flow cytometry

HUVECs and ARPE‐19 cells were seed on 6‐well plate at the density of 4 × 10^5^ cells per well for 24 h and were continued incubated with Cy5‐tFNAs‐DJ‐1‐saRNA, Cy5‐tFNAs, and Cy5‐DJ‐1‐saRNA for 12 h, respectively. Then, samples meeting the requirements of flow cytometry were collected. Flow cytometry (Attune NxT, ThermoFisher Scientific, USA) was used to spot the intracellular fluorescence intensity at 12 h.

### 2.4 RNA sequencing, data processing and bioinformatics analysis

ARPE‐19 mRNA sequencing (mRNA‐seq) were performed by the LC Bio Corporation (Hangzhou, China). In brief, raw reads, which were generated from the Illumina HiSeq platform, were checked, and cleaned with FastQC (v 0.11.2) and Trimmomatic (v 0.36), respectively. Subsequently, alignment and gene expression analysis were implemented using HISAT2 (version 2.1.0) and StringTie (v 1.3.3b). Furthermore, we used DESeq2 package24 to analyse differential gene expression in the ARPE‐19 cells between the control group, H_2_O_2_ group, and tFNAs‐DJ‐1‐saRNA group.^24^ In the H_2_O_2_ group, ARPE‐19 cells were cultured in DMEM/F‐12 containing 400 μM H_2_O_2_ for 6 h. In the tFNAs‐DJ‐1‐saRNA group, ARPE‐19 cells were cultured with 125 nM tFNAs‐DJ‐1‐saRNA for 24 h after exposing to 400 μM H_2_O_2_ for 6 h. Genes with absolute value of logFC (log_2_ fold change) > 1 and an adjusted *p*‐value of 0.05 were identified as differentially expressed genes (DEGs) and visualized by volcano plots and heatmaps using R4.3.1. Gene ontology (GO) pathway enrichment analysis for DEGs was then performed using culsterProfiler package and terms with *q*‐value <0.05 were presented.^25^

### 2.5 Cell viability measurements

HUVECs and ARPE‐19 cells were cultured on a 96‐well plate at the density of 5 × 10^4^ cells per well and treated as described at cell culture and treatment. The absorbance of the samples was measured at 450 nm by micro‐plate reader according to the instructions of the Cell Counting Kit‐8 (CCK‐8, Yeasen Biotechnology, China).

### 2.6 Real‐time fluorescence quantitative polymerase chain reaction analysis

The total RNA was isolated and purified with FastPure Cell/Tissue Total RNA Isolation Kit (Vazyme Biotechnology, China) according to the manufacturer's instructions. Then, HiScript III Reverse Transcriptase (Vazyme Biotechnology, China) was used to reverse transcription. All target mRNAs were amplified by real‐time fluorescence quantitative PCR (RT‐PCR) using SYBR Green I PCR Master Mix. Table S2 lists the corresponding primers. All BLAST search designs with GAPDH amplification as the control.

### 2.7 Western blotting

Total cell proteins were collected from the HUVECs and ARPE‐19 cells with M‐PER Mammalian Protein Extraction Reagent (ThermoFisher Scientific, USA) containing protease inhibitors. Protein concentrations were determined by the BCA Protein Quantitation Assay Kit (KeyGEN, Nanjing, China) using protein standard solution as a standard. Samples of supernatants containing 20 μg protein were heated to 100°C for 10 min and isolated with 10% or 12% SDS‐PAGE gel and transferred to polyvinylidene difluoride filter (PVDF) membrane (Millipore, Merck, Germany). Then, the PVDF membrane was blocked with Blocking Buffer (Quick Block Blocking Buffer for Western Blot, Beyotime, Shanghai, China) under ambient temperature for 2 h. Incubated the membrane with the diluted primary antibody at 4°C overnight, PARK7/DJ1 (1:10,000; ab18257, Abcam), Caspase‐3 (1:500; ab13847, Abcam), Bax (1:1000; ab32503, Abcam), Bcl‐2 (1:500; ab196495, Abcam), Erk1/2 (1:10,000; ab184699, Abcam), p‐Erk1/2 (1:5000; ab278538, Abcam), Elk‐1 (1:500; ab32106, Abcam), p‐Elk‐1 (1:500; ab218133, Abcam), Akt (1:500; ab8805, Abcam), p‐Akt (1:500; ab8933, Abcam), Nrf2 (1:500; ab137550, Abcam) and HO‐1 (1:2000; ab189491, Abcam). GAPDH (1:500; ab8245, Abcam) was used as an internal control. Incubated the membrane with anti‐rabbit IgG, Horseradish peroxidase‐linked antibody (1: 2000, ab205718, Abcam) under ambient temperature for 60 min, and visualized using an enhanced chemiluminescence system (ProteinSimple, USA).

### 2.8 Immunofluorescence

Cells were plated on dishes and incubated with Cy5‐tFNAs‐DJ‐1‐saRNA, Cy5‐tFNAs, or Cy5‐DJ‐1‐saRNA for 24 h. Cells were then washed thrice with phosphate buffer solution (PBS), fixed with paraformaldehyde for 15 min at 4°C, and incubated with fluorescein isothiocyanate (FITC)‐phalloidine and 4′,6‐diamidino‐2‐phenylindole for cytoskeleton staining and nucleus staining, respectively. Finally, the immunofluorescence samples were observed with a laser scanning confocal microscope.

### 2.9 Enzyme‐linked immunosorbent assay

Total cell proteins were isolated with M‐PER Mammalian Protein Extraction Reagent as described above. The quantitative measurement of human DJ‐1/PARK‐7 was analysed using Human PARK7 Enzyme‐Linked Immunosorbent Assay (ELISA) Kit (JL14590, Jianglai Biotechnology, China) according to the manufacturer's instructions. Absorbance from each sample was measured in duplicate using a micro‐plate reader, at dual wavelengths of 450/540 nm.

### 2.10 ROS level detection

Detection of ROS in the H_2_O_2_‐exposed cells was performed by flow cytometry. DCFH‐DA detection kit (Yeasen Biotechnology, China) was used for the detection of intracellular ROS. The measurements were carried out after 30 min of incubation with 10 mM of DCFH‐DA in DMEM without FBS at 37°C. The cells were collected, washed with cold PBS, and suspended in PBS, and subjected to flow cytometric analysis. The excitation was done at a wavelength of 488 nm. The measured intensity is proportional to the concentration of ROS inside the cells.

### 2.11 Transmission electron microscopy

According to the standard procedures, cells were obtained, prefixed with a 3% glutaraldehyde, post‐fixed in 1% osmium tetroxide, dehydrated in series acetone and embedded. The sections were evaluated using a TEM JEM‐1400FLASH.

### 2.12 Mitochondrial inner membrane potential (ΔΨm) assessment

The mitochondrial membrane potential (Δψm) was detected using JC‐10 mitochondria staining (Yeasen Biotechnology, China). Briefly, cells were incubated with JC‐10 (20 μM) at 37°C for 30 min and rinsed three times with PBS. Observations were made using flow cytometry and fluorescence microscope. JC‐1 monomer (green) fluorescence was observed by excitation at 488 nm and examination of the emission at 530 nm. JC‐10 aggregate (red) fluorescence was observed by examination of the emission at 590 nm. The ratio of JC‐10 aggregate to monomer intensity for each region was calculated.

### 2.13 Cellular bioenergetics measurements

Oxygen consumption rate (OCR) was assessed using the Seahorse XFe24 Extracellular Flux Analyser (Agilent Technologies, CA) with a real time ATP Test Kit (Seahorse Biosciences). ARPE‐19 and HUVEC cells were seeded into Seahorse XFe24 V7 microplates at a density of 5 × 10^3^ cells/well. The sensor cartridges in Seahorse XF calibrant were hydrated at 37°C in a non‐CO_2_ incubator overnight. And the culture medium was substituted with Agilent Seahorse XF DMEM medium supplemented with 1 mM of sodium pyruvate, 2 mM of glutamine and 1 mM D‐glucose. The cells were then placed in an incubator at 37°C for 60 min prior to test, followed by washing with Seahorse buffer for subsequent experimental operations. Meanwhile, 1.5 μM oligomycin and 0.5 μM rotenone/antimycin A (Rot/AA) were injected to assess the real‐time ATP rate, OCR, extra cellular acidification rate (ECAR) and proton efflux rate (PER). Cell concentrations were determined using a cell counter and real‐time ATP rate, OCR, ECAR and PER were normalized by total cell concentration.

### 2.14 Statistical analysis

All data were analysed using GraphPad Prism 8.0 software, and were presented as mean ± standard deviation (SD). After confirming the data normality, student's *t*‐test was applied to compared means of two groups. One‐way analysis of variance (ANOVA) was used for multiple‐group comparisons. *P*‐value <0.05 were considered significant.

## 3 RESULTS

### 3.1 Synthesis and characterization of tFNAs‐DJ‐1‐saRNA

**FIGURE 1:**
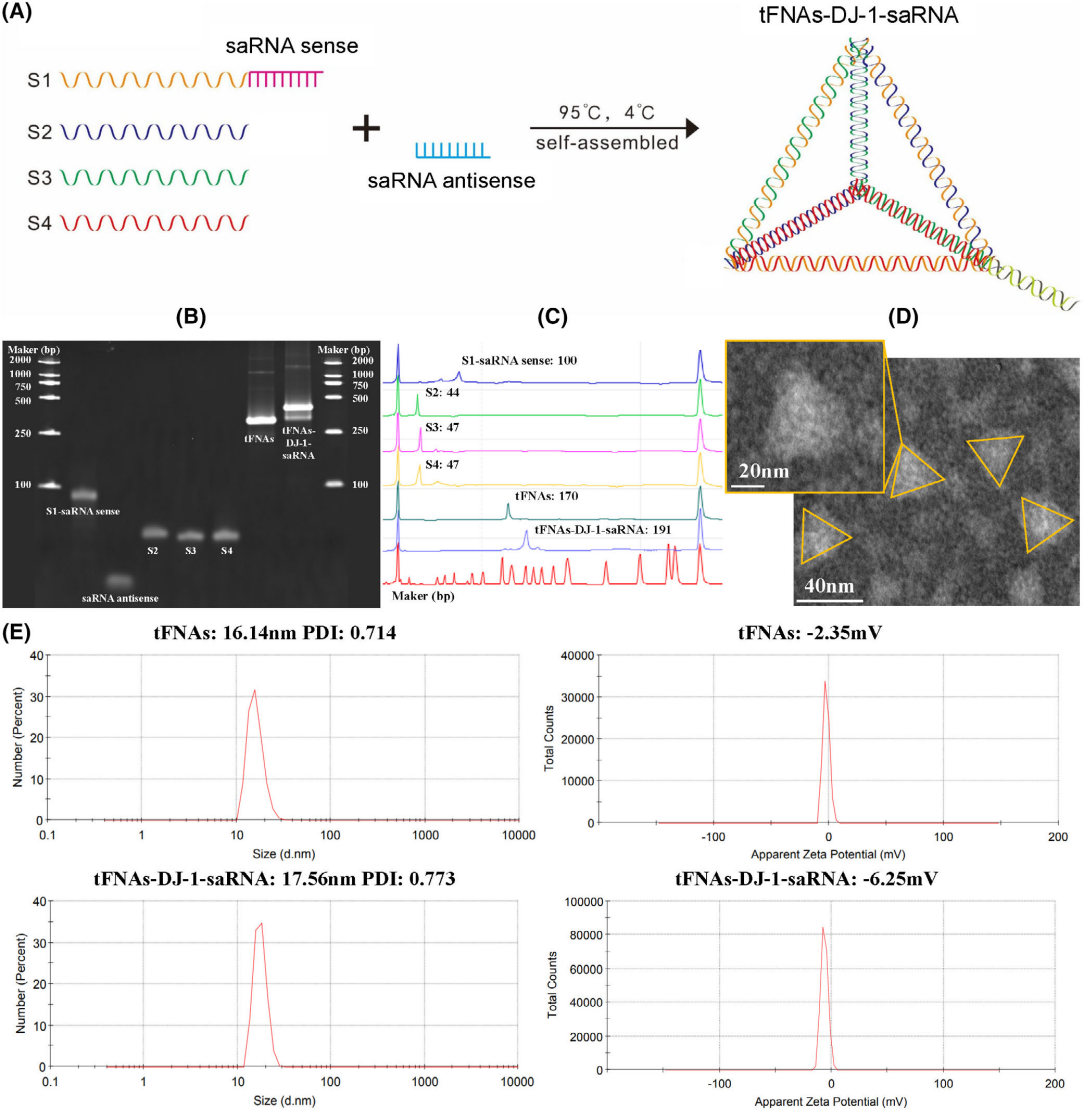
Preparation and characterization of tFNAs‐DJ‐1‐saRNA. (A) Schematic diagram of the preparation of tFNAs‐DJ‐1‐saRNA. (B) Confirmation of the successful synthesis of tFNAs and tFNAs‐DJ‐1‐saRNA by native‐polyacrylamide gel electrophoresis. Lane 1, S1‐saRNA sense; Lane 2, saRNA‐antisense; Lane 3, S2; Lane 4, S3; Lane 5, S4; Lane 6, tFNAs; Lane 7, tFNAs‐DJ‐1‐saRNA. (C) The successful generation of the tFNAs and tFNAs‐DJ‐1‐saRNA was verified by the high‐performance capillary electrophoresis. (D) Transmission electron microscopic image of tFNAs‐DJ‐1‐saRNA. Scale bars are 40 nm for transmission electron microscopy. (E) Particle size and zeta potential results for the tFNAs and tFNAs‐DJ‐1‐saRNA measured by dynamic light scattering.

Four different saRNAs targeting DJ‐1 were designed, and their potency to improve DJ‐1 expression were validated by RT‐qPCR and ELISA in HUVECs and ARPE‐19 cells. After transfection with 4 different saRNAs for 24 h with the help of transfection reagent, saRNA 3 was finally selected for the synthesis of tFNAs‐DJ‐1‐saRNA in the following studies (Figure S1). tFNAs was easily self‐assembled from four specifically designed ssDNAs strands (S1, S2, S3 and S4, Table S1) through a simple annealing process in accordance with previous studies.^26^ In this study, we modified DJ‐1‐saRNA sense to the 3′ end of S1 in a similar way as previous study to form a new S1 (S1‐saRNA sense, Table S1),^27^ and then synthesized tFNAs‐DJ‐1‐saRNA in the same steps as tFNAs. Schematic diagram of the synthesis process of the tFNAs‐DJ‐1‐saRNA was shown in Figure 1A. Next, we used 8% PAGE to verify the successful synthesis of tFNAs‐DJ‐1‐saRNA. Slower migration of tFNAs‐DJ‐1‐saRNAs (lane 7) compared to S1‐saRNA sense (lane 1), saRNA antisense (lane 2), ssDNAs (lanes 3–5) and tFNAs (lane 6) were observed (Figure 1B). Results of HPCE also confirmed the successful synthesis of tFNAs‐DJ‐1‐saRNA in 191 bp (Figure 1C). The TEM image demonstrated that tFNAs‐DJ‐1‐saRNA had a triangle‐like shape on the two‐dimensional plane (Figure 1D), which is in accordance with previous studies.^27^ In addition, the particle size of tFNAs was approximately 16.14 nm, while the size of tFNAs‐DJ‐1‐saRNA was approximately 17.56 nm, as measured by DLS. And the zeta‐potential of tFNAs‐DJ‐1‐saRNA (approximately −6.25 mV) was slightly lower than that of tFNAs (approximately −2.35 mV) (Figure 1E). This indicated that the structural stability of tFNAs‐DJ‐1‐saRNA was comparable to that of tFNAs. Taken together, these results suggested that DJ‐1‐saRNA was successfully loaded onto tFNAs.

### 3.2 Cellular uptake of tFNAs‐DJ‐1‐saRNA and cellular DJ‐1 expression

**FIGURE 2:**
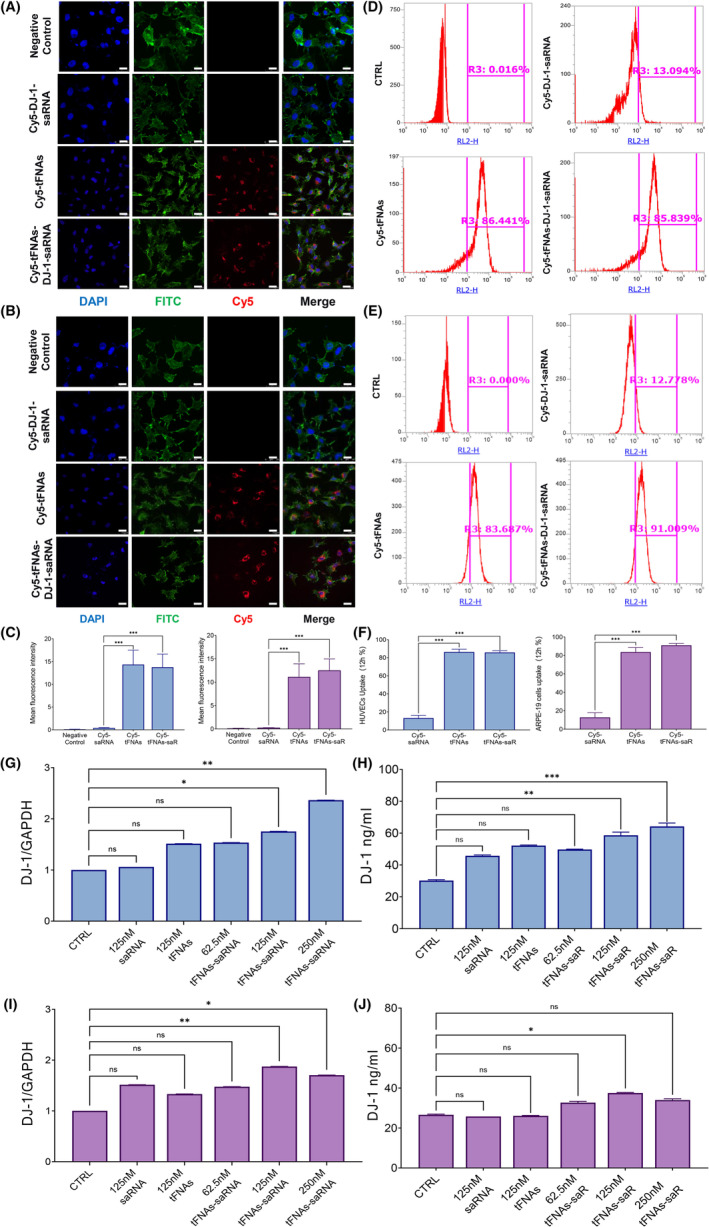
(A, B) Cellular uptake of Cy5‐DJ‐1‐saRNA, Cy5‐tFNAs, and Cy5‐tFNAs‐DJ‐1‐saRNA by human umbilical vein endothelial cells (HUVECs) (top) and ARPE‐19 cells (bottom) for 24 h as determined by immunofluorescence staining (Cy5‐DJ‐1‐saRNA, Cy5‐tFNAs, and Cy5‐tFNAs‐DJ‐1‐saRNA: red; nucleus: blue; cytoskeleton: green). Scale bars are 20 μm. (C) Mean fluorescence intensity of Cy5‐tFNAs and Cy5‐tFNAs‐DJ‐1‐saRNA was significantly higher than that of Cy5‐DJ‐1‐saRNA in HUVECs (left) and ARPE‐19 (right) cells for 24 h. Data are presented as mean ± SD (*n* = 3). (D, E) Quantitative detection and analysis of the uptake rate of DJ‐1‐saRNA, tFNAs and tFNAs‐DJ‐1‐saRNA in HUVECs (top) and ARPE‐19 cells (bottom) for 12 h using flow cytometry. (F) The uptake rate of tFNAs and tFNAs‐DJ‐1‐saRNA was significantly higher than that of DJ‐1‐saRNA in HUVECs and ARPE‐19 cells for 12 h. Data are presented as mean ± SD (*n* = 4). (G, H) The expression of DJ‐1 mRNA (left) and protein (right) in HUVECs treated with different concentrations of DJ‐1‐saRNA, tFNAs, and tFNAs‐DJ‐1‐saRNA for 24 h. Data are presented as mean ± SD (*n* = 3). (I, J) The expression of DJ‐1 mRNA (left) and protein (right) in ARPE‐19 cells treated with different concentrations of DJ‐1‐saRNA, tFNAs, and tFNAs‐DJ‐1‐saRNA for 24 h. Data are presented as mean ± SD (*n* = 3). Statistical analysis: the ANOVA test was applied; no significance (ns): *p* ≥ 0.05; *p < 0.05; **p < 0.01; ***p < 0.001.

To investigate the potential protective effects of tFNAs‐DJ‐1‐saRNA in AMD and DR, HUVECs and ARPE‐19 cells were used in this study. Firstly, the intracellular uptake of tFNAs‐DJ‐1‐saRNA was evaluated using flow cytometry and immunofluorescence (Figure 2). Observations from confocal laser scanning microscopy showed that most cells stained with FITC‐phalloidine exhibited red fluorescence after being incubated with Cy5‐tFNAs‐DJ‐1‐saRNA and Cy5‐tFNAs (Figure 2A–C). However, no significant fluorescence was observed with Cy5‐DJ‐1‐saRNA. The flow cytometry results showed that over 80% of the cells could ingest Cy5‐tFNAs‐DJ‐1‐saRNA or Cy5‐tFNAs after 12 h incubation (Figure 2D–F). However, the uptake rate of Cy5‐DJ‐1‐saRNA was relatively low at around 10%. These findings indicated that tFNAs could facilitate the uptake of DJ‐1‐saRNA in both HUVECs and ARPE‐19 cells.

Then, we used the RT‐PCR, western blotting and ELISA to measure the DJ‐1 expression after the tFNAs‐DJ‐1‐saRNA incubation (Figures 2G–J and S3). The results showed that 125 nM tFNAs‐DJ‐1‐saRNA significantly increased the level of DJ‐1 mRNA and protein in both HUVECs and ARPE‐19 cells. Meanwhile, DJ‐1‐saRNA had no significant effects on DJ‐1 expression. Thus, tFNAs may serve as an efficient delivery system for DJ‐1‐saRNA, facilitating the upregulation of target gene expression and the consequent manifestation of relevant biological effects.

### 3.3 tFNAs‐DJ‐1‐saRNA protect against oxidative injury

To study the antioxidant effects of tFNA‐DJ‐1 saRNAs, HUVECs and ARPE‐19 were exposed to different concentration of H_2_O_2_ (100, 200, 300, 400, 500, 600, 700 and 800 μM) for 6, 24 and 36 h to stimulate oxidative stress, respectively. CCK8 assay indicated that although ARPE‐19 seemed to have a better resistance to H_2_O_2_‐induced oxidative stress, the viability of HUVECs and APRE‐19 cells both decreased as the concentration and length of time to H_2_O_2_ exposure increased (Figure S2). The cell viability of HUVECs and ARPE‐19 cells decreased to around 50% after co‐culturing with 200 and 400 μM H_2_O_2_ for 6 h, respectively. We also explore HUVECs and ARPE‐19 to different concentration of tFNAs‐DJ‐1‐saRNA. Data suggested that 0–500 nM tFNAs‐DJ‐1‐saRNA (0, 62.5, 125, 250, 500 nM) had no significant effects on the cell viability (Figure S4).

**FIGURE 3:**
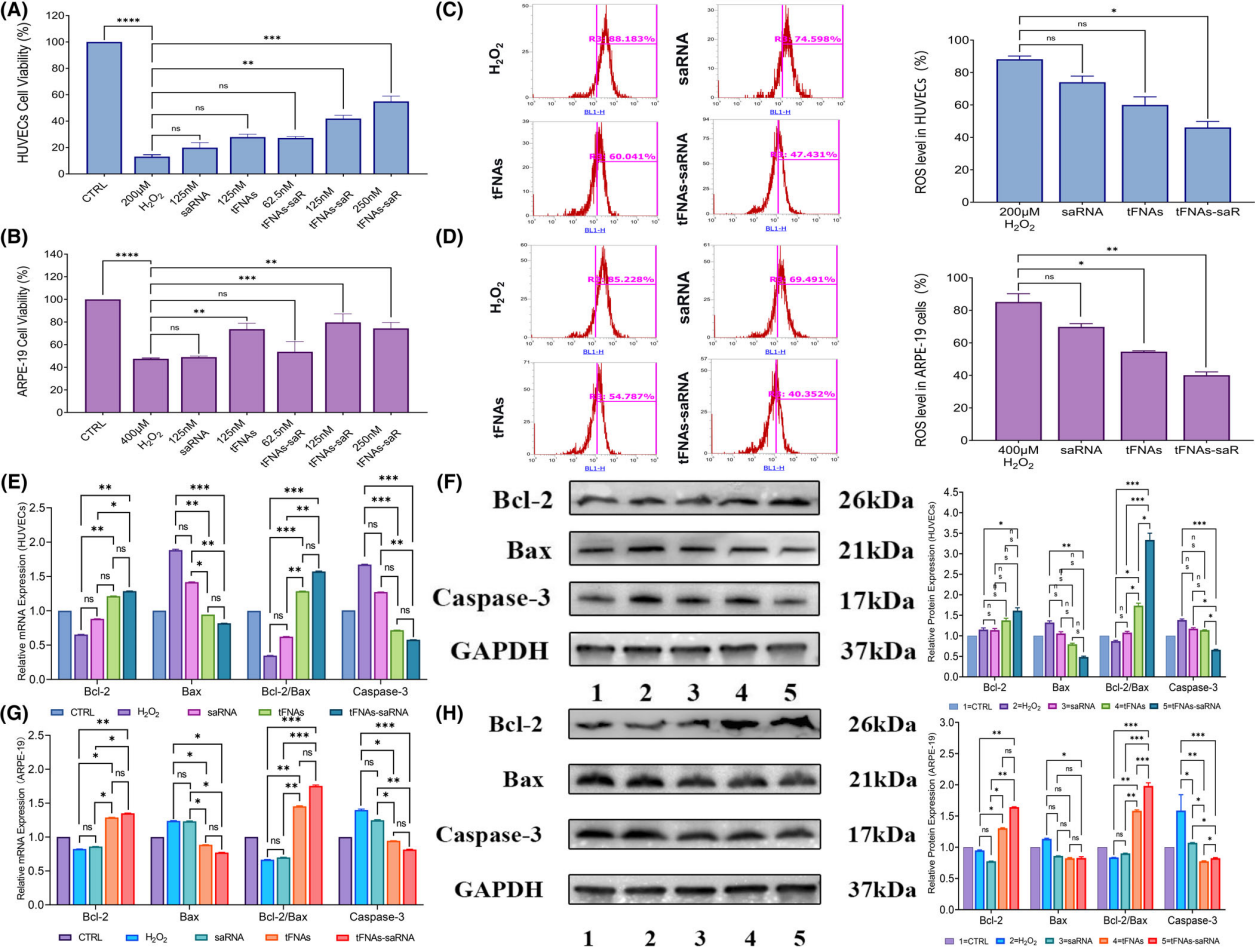
(A, B) The cell viability of human umbilical vein endothelial cells (HUVECs) (top) and ARPE‐19 cells (bottom) was evaluated after exposure to H_2_O_2_, DJ‐1‐saRNA, tFNAs, tFNAs‐DJ‐1‐saRNA of different concentrations. Data are presented as mean ± SD (*n* = 6). (C, D) Quantitative detection and analysis of the intracellular ROS levels after different treatment regimes in HUVECs (top) and ARPE‐19 cells (bottom) using flow cytometry. Data are presented as mean ± SD (*n* = 3). (E, G) Quantification of Bcl‐2, Bax and Caspases‐3 mRNA expression in different treated HUVECs (top) and ARPE = 19 cells (bottom) by real‐time quantitative PCR (RT‐qPCR). Data are presented as mean ± SD (*n* = 3). (F, H) The expressions of Bcl‐2, Bax, and Caspases‐3 in different treated HUVECs (top) and ARPE = 19 cells (bottom) were detected by Western blot. The expression levels of related proteins in the above mitochondrial apoptosis pathway in different treated HUVECs (top) and ARPE = 19 cells (bottom) were quantitatively analysed. All data are performed using one‐way ANOVA followed by Bonferroni correction for multiple comparisons. Data are presented as mean ± SD (*n* = 3). Statistical analysis: no significance (ns): *p* ≥ 0.05; *p < 0.05; **p < 0.01; ***p < 0.001. GAPDH was used as an internal control.

We chose a concentration of 200 μM H_2_O_2_ for HUVECs incubation and 400 μM for ARPE‐19 cells in the subsequent experiments. tFNAs‐DJ‐1‐saRNA were simultaneously added to assess its ability to improve cell viability. The results showed that both 125 and 250 nM tFNAs‐DJ‐1‐saRNA significantly improved the cell viability of HUVECs, with 250 nM tFNAs‐DJ‐1‐saRNA having the most significant effect. In contrast, DJ‐1‐saRNA and tFNAs alone did not affect cell viability of HUVECs (Figure 3A). Furthermore, we observed that 125 nM tFNAs‐DJ‐1‐saRNA had the best effect on improving cell viability of ARPE‐19 cells. Both 125 and 250 nM tFNAs‐DJ‐1‐saRNA, as well as 125 nM tFNAs enhanced the survival of APRE‐19 cells (Figure 3B). These results were consistent with the antioxidant effects of tFNAs reported in previous studies.^21^, ^28^ Therefore, we treated HUVECs and ARPE‐19 cells with 250 and 125 nM concentrations of different drugs (DJ‐1‐saRNA, tFNAs and tFNAs‐DJ‐1‐saRNA), and then determined the cellular ROS expression of HUVECs and ARPE‐19 cells after the treatment, respectively. Flow cytometric analysis revealed that tFNAs‐DJ‐1‐saRNA treatment could effectively lower the cellular ROS level in both HUVECs and ARPE‐19 cells (Figure 3C, D).

Furthermore, we accessed the levels of some critical apoptosis‐related proteins to further investigate the effect of tFNAs‐DJ‐1‐saRNA on the apoptosis of HUVECs and ARPE‐19 cells. The Bcl‐2 family is the primary family of proteins related to apoptosis, and both the pro‐apoptotic protein Bax and the anti‐apoptotic protein Bcl‐2 are members of this family. Caspase‐3 is the key molecule associated with apoptosis in the Caspase family.^29^ This is the convergence point for different pro‐apoptotic signals during apoptosis, triggering the cascades that are associated with mitochondria‐dependent and death‐receptor‐mediated signalling pathways. As expected, H_2_O_2_ incubation significantly decreased the expression of Bcl‐2, and increased the expression of Bax and Caspase 3 in HUVECs and ARPE‐19 cells not only in the mRNA level but also in the protein level (Figure 3E–H). In HUVECs, the ratio of Bcl‐2/Bax in the 250 nM tFNAs‐DJ‐1‐saRNA group was significantly higher than that in other groups. In comparison, the expression of Caspase3 was conspicuously lower than that in the other groups. Besides, the results of apoptosis‐related proteins expression detected by RT‐PCR were consistent with western blot. Meanwhile, we also found that both 125 nM tFNAs‐DJ‐1‐saRNA and tFNAs could significantly improve the ratio of Bcl‐2/Bax and downregulate the expression of Caspase3 in ARPE‐19 cells. Take together, the results confirmed the protective effects of tFNAs‐DJ‐1‐saRNA against apoptosis during oxidative stress in both HUVECs and APRE‐19 cells, and indicated that tFNAs might also possess anti‐apoptosis effects in ARPE‐19 cells.

### 3.4 tFNAs‐DJ‐1‐saRNA preserved mitochondrial structure and function

**FIGURE 4:**
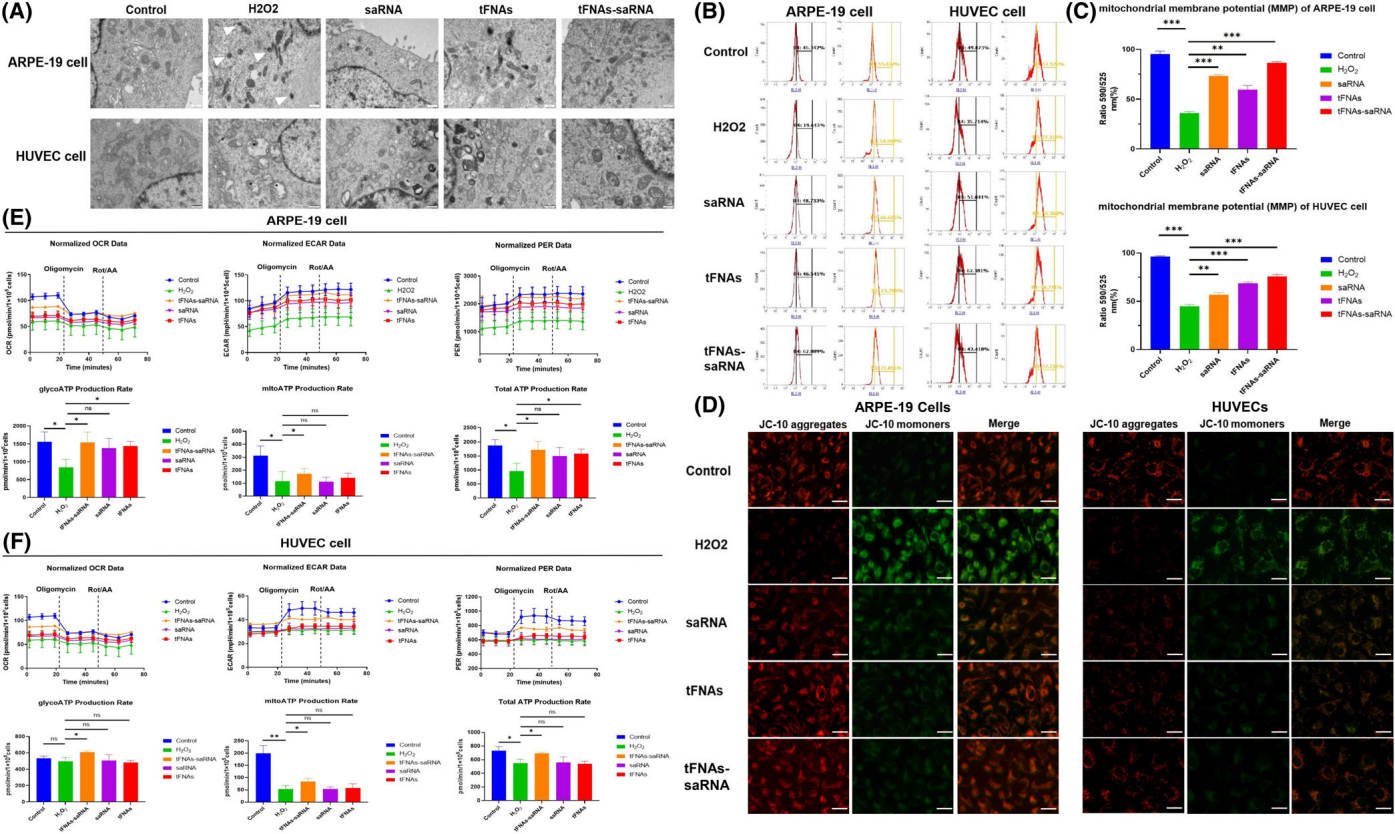
(A) Transmission electron microscopy images of the overall and mitochondrial structure in human umbilical vein endothelial cells (HUVECs) after exposure to H_2_O_2_, DJ‐1‐saRNA, tFNAs, tFNAs‐DJ‐1‐saRNA of different concentrations. Shrunken mitochondria (white arrowheads) in ARPE‐19 cells and cytoplasmic and organelle swelling (asterisk) in HUVECs after H_2_O_2_ treatment were observed. (B, C, D) Detection of mitochondrial membrane potential with JC‐10 dye. Cells from each group were stained with JC‐10 reagent, and then analysed by immunofluorescence and flow cytometry. Scale bars are 500 nm. Data are presented as mean ± SD (*n* = 3). (E, F) Seahorse cell mito stress test was performed to measure OCR in ARPE‐19 and HUVECs following a sequential addition of inhibitors of mitochondrial function: oligomycin and a combination of rotenone and antimycin A. Oxygen consumption rate (OCR), extracellular acidification rate (ECAR) and proton excretion rate (PER) are measured using the Seahorse XFe24 V7 Extracellular Flux Analyser. GlycoATP production rate, mitoATP production rate and total production rate were calculated. Data are presented as means ± SD from three independent experiments. Data are presented as mean ± SD (*n* = 3). Statistical analysis: the ANOVA test was applied; no significance (ns): *p* ≥ 0.05; *p < 0.05; **p < 0.01; ***p < 0.001.

TEM allows detailed identification of ultrastructural changes of cells, thus was used for further analysis. We observed cytoplasmic and organelle swelling, and shrunken mitochondria in ARPE‐19 cells after H_2_O_2_ treatment (Figure 4A). The alteration of mitochondria in H_2_O_2_ group were particularly prominent, as mitochondria became smaller and hyperchromatic, the cristae of mitochondria were destroyed, and outer mitochondrial membrane were ruptured. All these morphological changes are hallmarks of ferroptosis. In HUVECs affected by H_2_O_2_, some mitochondria change into small vesicular structures under TEM, which are features of apoptosis. tFNAs‐DJ‐1‐saRNA treatment significantly alleviate the pathological changes of ARPE‐19 and HUVECs (Figure 4A).

In view of the significant changes in the mitochondria of H_2_O_2_‐treated HUVECs and ARPE‐19, we further assess the alterations in mitochondrial function. Mitochondria are essential organelles that are responsible for ATP production, redox signalling and cell death. First, we used JC‐10 probe to determine mitochondrial membrane potential. Uptake of JC‐10 into the mitochondrial matrix is driven by polarized Δψm and results in JC‐10 accumulation in cells with healthy mitochondria, and forms red fluorescent aggregation. While in apoptotic and necrotic cells, JC‐10 exists in monomeric form and stains cells green. The results of the flow cytometry analysis and immunofluorescence showed a significant decrease in the intensity of red fluorescent signal, and an increase in the green fluorescent monomeric signal after H_2_O_2_ incubation (Figure 4B–D). A successful rescue of red fluorescent signal with the treatment of tFNAs‐DJ‐1‐saRNA in both ARPE‐19 and HUVEC was also confirmed, which prove that tFNAs‐DJ‐1‐saRNA helps reduce H_2_O_2_‐induced cellular stress and cytotoxicity.

Further, we sought to determine whether metabolic reprogramming during H_2_O_2_ impacts stress response of ARPE‐19 and HUVEC. OCR, ECAR and PER are measured using the Seahorse XFe24 V7 Extracellular Flux Analyser. Our data showed that H_2_O_2_‐treated cells displayed decreased OCR/ECAR/PER, while tFNAs‐DJ‐1‐saRNA treatment restored the OCR/ECAR/PER (Figure 4E). ATP production from mitochondrial respiration and glycolysis was calculated from the OCR, ECAR and PER traces. Our results indicated that H_2_O_2_ reduced total ATP production in HUVECs, and the decline was mostly contributed by impaired ATP production from oxidative phosphorylation in the mitochondrial respiratory chain (Figure 4F). While in H_2_O_2_‐treated ARPE‐19, mitochondrial ATP production and glycolytic ATP production concomitantly decreased. Moreover, only tFNAs‐DJ‐1‐saRNA was confirmed to recover both glycoATP production and mitoATP production in HUVECs. tFNAs and DJ‐1‐saRNA addition solely had no significant effects on metabolic stress response of HUVECs. In H_2_O_2_‐incubated ARPE‐19 cells, tFNAs‐DJ‐1‐saRNA showed similar beneficial influence on the glycoATP production and mitoATP production. Glycolytic reserve was also found to be significantly higher after tFNAs treatment. Collectively, these results clearly demonstrate tFNAs‐DJ‐1‐saRNA not only help maintain the integrity of mitochondrial structure under oxidative stress, but also preserve cellular redox homeostasis and mitochondrial function.

### 3.5 tFNAs‐DJ‐1‐saRNA regulated Erk/Elk‐1 and Nrf2/HO‐1 pathway

**FIGURE 5:**
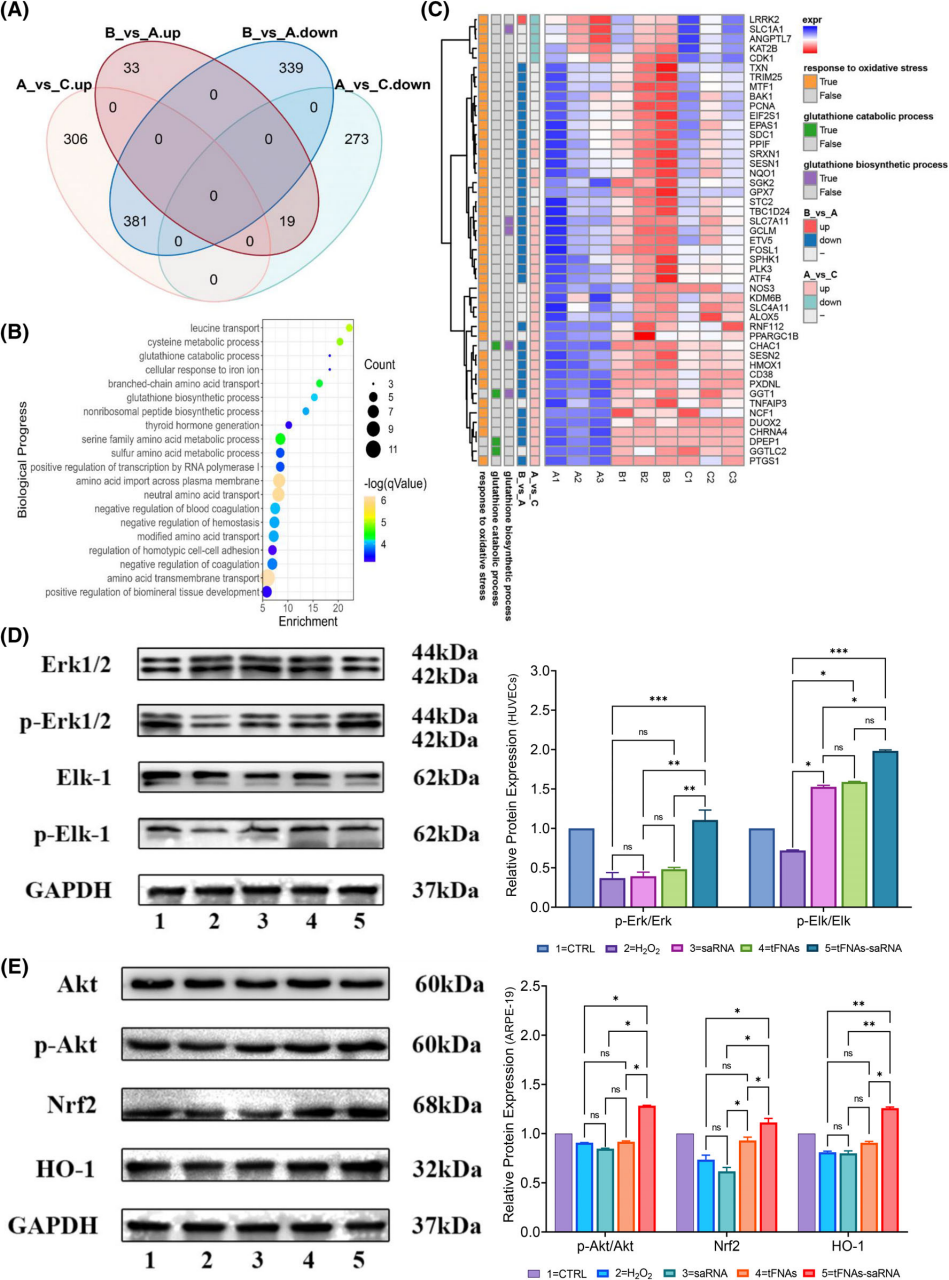
tFNAs‐DJ‐1‐saRNA promotes human umbilical vein endothelial cells (HUVECs) survival by regulating the Erk‐Elk signalling pathway. (A) The Venn plot showed that the expression of DEGs between H_2_O_2_ treatment (A) and the control (C) group, tFNAs‐DJ‐1‐saRNA (B) and H_2_O_2_ treatment (A) group. (B) The volcano plots showed enriched pathways. (C) The heatmap showed response to oxidative stress, glutathione biosynthetic process and glutathione catabolic process were enriched. (D) The expressions of Erk1/2, p‐Erk1/2, Elk‐1 and p‐Elk‐1 in different treated HUVECs were detected by Western blot. Data are presented as mean ± SD (*n* = 3). (E) The expressions of Akt, p‐Akt, Nrf2 and HO‐1 in different treated ARPE‐19 cells were detected by Western blot. All data are performed using one‐way ANOVA followed by Bonferroni correction for multiple comparisons and presented as mean ± SD (*n* = 3). Statistical analysis: no significance (ns): *p* ≥ 0.05; *p < 0.05; **p < 0.01; ***p < 0.001. GAPDH was used as an internal control.

Finally, we performed RNA sequencing to explore the DEGs in ARPE‐19 and HUVEC after H_2_O_2_ treatment. The gene expression profiles between H_2_O_2_ group (A) and the control group (C), tFNAs‐DJ‐1‐saRNA group (B) and H_2_O_2_ group (A) were compared. The Venn plot showed that a large number of up‐regulated genes (381) induced by H_2_O_2_ were down‐regulated after tFNAs‐DJ‐1‐saRNA treatment, and 19 down‐regulated genes induced by H_2_O_2_ were up‐regulated by tFNAs‐DJ‐1‐saRNA in ARPE‐19 (Figure 5A). The similar tendency was also noticed in HUVECs (Figure S5). All those genes displayed opposite expression tendency were included for GO pathway enrichment analysis, and the volcano plots showed top 20 enriched pathway (Figure 5B). Particularly, DEGs from the response to oxidative stress pathway is enriched according to the GO analysis. The glutathione biosynthetic process and glutathione catabolic process pathway which were closely related to ferroptosis were also enriched, which is consistent with our observations by TEM (Figure 5C). Then, we explore the potential role of tFNAs‐DJ‐1‐saRNA on oxidative stress‐related signalling pathway. Studies have reported that DJ‐1 can mediate cell survival by activating the extracellular signal‐regulated kinase (Erk) pathway and the downstream nuclear factor erythroid 2‐related factor 2 (Nrf2) pathway.^30^, ^31^ Our western blotting results also showed that the phosphorylation of Erk1/2 and its downstream transcription factor Elk‐1 was upregulated by 250 nM tFNAs‐DJ‐1‐saRNA in H_2_O_2_‐treated HUVECs (Figure 5D). On the other hand, 125 nM tFNAs‐DJ‐1‐saRNA could also efficiently promote the expression of the phosphorylation of Akt, Nrf2, and heme oxygenase‐1 (HO‐1) in ARPE‐19 cells (Figure 5E).

## 4 DISCUSSION

As global leading causes of blindness. There are disappointingly few therapeutic options for AMD and DR.^32^ Intravitreal injection of drugs targeting vascular endothelial growth factor (VEGF) remained as the principal non‐destructive management for DR and the wet form of AMD. More therapeutic treatments are urgently needed for the benefits of the AMD and DR patients.

The importance of oxidative stress in the pathophysiology of DR and AMD was widely recognized, thus endorsing antioxidant therapy as the main target for their management.^3^, ^33^ DJ‐1 protein, known as an oxidative stress sensor, plays a major role in anti‐oxidant process. DJ‐1 is up‐regulated in response to oxidative insult serving as a cellular feedback mechanism to confer antioxidative defence during various physiological and pathological processes.^34^ In this study, we identified overexpressing DJ‐1 protein as a novel strategy for AMD and DR treatment.

Traditionally, researches exploring DR or AMD treatment mainly focus on the inhibition of key factors, such as VEGF, APE/REF‐1 and inflammatory factors.^35^ Gene overexpression therapy are rarely involved because of the lack of safe and effective gain‐of‐function approach. Clinically, viral‐based systems to deliver exogenous genes were the major choices for gene overexpression. However, it was based on a constructed exogenous DNA for ectopic expression which has detrimental effects.^36^ RNAa offers a new option for gene upregulation by selectively enhancing the transcription of an endogenous gene,^37^ while avoid the tedious properties of viral‐based systems such as endangering host genome integrity and undesired immunological consequences. To our knowledge, it was the first time that saRNAs were used in retinal or any other ocular diseases. Moreover, we initiated the attempt to employ tFNAs to deliver and promote the bioavailability of saRNAs.

In conclusion, we established a simple and efficient delivery system by modifying DJ‐1‐saRNA onto tFNAs. We found that tFNAs could penetrate the cell plasma membrane without any transfection agent, transfer DJ‐1‐saRNA into damaged HUVECs and ARPE‐19 cells safely and efficiently, and significantly increased the expression of DJ‐1 protein. The preliminary in vitro assessments showed that tFNAs served as an ideal delivery platform for DJ‐1‐saRNA with excellent biocompatibility. Then, we observed that H_2_O_2_ incubation decreased cell viability in a concentration/time‐dependent manner. When 250 and 125 nM tFNAs‐DJ‐1‐saRNA were respectively added into the cell media in HUVECs and ARPE‐19 cells, a significant improvement of cell viability was observed. Furthermore, tFNAs‐DJ‐1‐saRNA could inhibit the expression of pro‐apoptosis proteins, and ROS production and restore the function and structure of mitochondrions during oxidative stress, while DJ‐1‐saRNA had no effect. The results demonstrated that protective effects of DJ‐1‐saRNA was prominent only when it was loaded onto tFNAs. To explore the possible molecular mechanism of antioxidant effects of the tFNA‐DJ‐1‐saRNA, we evaluated the Erk and Nrf2 pathway. Western blotting results confirmed that tFNA‐DJ‐1‐saRNA could enhance the phosphorylation of Erk, Elk and Akt, increase the level of Nrf2 and HO‐1, thus activating the Erk and Nrf2 pathway. Based on these results, we believe that tFNAs represent a promising saRNAs delivery vehicle, and provides novel therapeutic options for the management of AMD and DR. However, our research only focused on cell biological effects in vitro, the confirmation of antioxidant effects of tFNAs‐DJ‐1‐saRNA within animals are needed in the future.

## AUTHOR CONTRIBUTIONS

Qiaowei Wu and Jingyi Zhu contributed equally to this work. Study conception and design: Yanping Song, Ming Yan, Jingyi Zhu, Yunfeng Lin, Delun Luo. Acquisition of data: Qiaowei Wu, Xianggui Zhang, Xiaoxiao Xu. Analysis and interpretation of data: Jingyi Zhu, Qiaowei Wu, Xianggui Zhang, Delun Luo.

## CONFLICT OF INTEREST STATEMENT

The authors declare no conflicts of interest.

## Supporting information


**Data S1.** Supporting Information.

**Table S1.** Base sequence of each ssDNA and saRNA.

**Table S2.** The sequences of PCR primers.

## Data Availability

The data that support the findings of this study are available on request from the corresponding author. The data are not publicly available due to privacy or ethical restrictions.

## References

[1] Nishimura Y , Hara H , Kondo M , Hong S , Matsugi T . Oxidative stress in retinal diseases. Oxid Med Cell Longev. 2017;2017:4076518. 28424744 10.1155/2017/4076518 PMC5382422

[2] Hammes H . Diabetic retinopathy: hyperglycaemia, oxidative stress and beyond. Diabetologia. 2018;61(1):29‐38. 28942458 10.1007/s00125-017-4435-8

[3] Cecilia O‐M , José Alberto C‐G , José N‐P , et al. Oxidative stress as the Main target in diabetic retinopathy pathophysiology. J Diabetes Res. 2019;2019:8562408. 31511825 10.1155/2019/8562408 PMC6710812

[4] Zhang L , Wang J , Wang J , Yang B , He Q , Weng Q . Role of DJ‐1 in immune and inflammatory diseases. Front Immunol. 2020;11(994). 10.3389/fimmu.2020.00994 PMC7308417 32612601

[5] Lev N , Ickowicz D , Melamed E , Offen D . Oxidative insults induce DJ‐1 upregulation and redistribution: implications for neuroprotection. Neurotoxicology. 2008;29(3):397‐405. 18377993 10.1016/j.neuro.2008.01.007

[6] Zeng J , Zhao H , Chen B . DJ‐1/PARK7 inhibits high glucose‐induced oxidative stress to prevent retinal pericyte apoptosis via the PI3K/AKT/mTOR signaling pathway. Exp Eye Res. 2019;189:107830. 31593688 10.1016/j.exer.2019.107830

[7] Wang W , Zhao H , Chen B . DJ‐1 protects retinal pericytes against high glucose‐induced oxidative stress through the Nrf2 signaling pathway. Sci Rep. 2020;10(1):2477. 32051471 10.1038/s41598-020-59408-2 PMC7016111

[8] Bonilha VL , Bell BA , Rayborn ME , et al. Loss of DJ‐1 elicits retinal abnormalities, visual dysfunction, and increased oxidative stress in mice. Exp Eye Res. 2015;139:22‐36. 26215528 10.1016/j.exer.2015.07.014 PMC4573318

[9] Upadhyay M , Milliner C , Bell BA , Bonilha VL . Oxidative stress in the retina and retinal pigment epithelium (RPE): role of aging, and DJ‐1. Redox Biol. 2020;37:101623. 32826201 10.1016/j.redox.2020.101623 PMC7767746

[10] Kim RH , Smith PD , Aleyasin H , et al. Hypersensitivity of DJ‐1‐deficient mice to 1‐methyl‐4‐phenyl‐1,2,3,6‐tetrahydropyrindine (MPTP) and oxidative stress. Proc Natl Acad Sci. 2005;102(14):5215‐5220. 15784737 10.1073/pnas.0501282102 PMC555037

[11] Zhou J , Zhang L , Wang M , et al. CPX targeting DJ‐1 triggers ROS‐induced cell death and protective autophagy in colorectal cancer. Theranostics. 2019;9(19):5577‐5594. 31534504 10.7150/thno.34663 PMC6735393

[12] De Miranda BR , Rocha EM , Bai Q , et al. Astrocyte‐specific DJ‐1 overexpression protects against rotenone‐induced neurotoxicity in a rat model of Parkinson's disease. Neurobiol Dis. 2018;115:101‐114. 29649621 10.1016/j.nbd.2018.04.008 PMC5943150

[13] Bulcha JT , Wang Y , Ma H , Tai PWL , Gao G . Viral vector platforms within the gene therapy landscape. Signal Transduct Target Ther. 2021;6(1):53. 33558455 10.1038/s41392-021-00487-6 PMC7868676

[14] Zhou LY , He ZY , Xu T , Wei YQ . Current advances in small activating RNAs for gene therapy: principles, applications and challenges. Curr Gene Ther. 2018;18(3):134‐142. 29921205 10.2174/1566523218666180619155018

[15] Ghanbarian H , Aghamiri S , Eftekhary M , Wagner N , Wagner K‐D . Small activating RNAs: towards the development of new therapeutic agents and clinical treatments. Cells. 2021;10:591. 33800164 10.3390/cells10030591 PMC8001863

[16] Kwok A , Raulf N , Habib N . Developing small activating RNA as a therapeutic: current challenges and promises. Ther Deliv. 2019;10(3):151‐164. 30909853 10.4155/tde-2018-0061

[17] Zhang T , Tian T , Lin Y . Functionalizing framework nucleic‐acid‐based nanostructures for biomedical application. Adv Mater. 2022;34(46):e2107820. 34787933 10.1002/adma.202107820

[18] Tetrahedral framework nucleic acids deliver antimicrobial peptides with improved effects and less susceptibility to bacterial degradation. Nano Lett. 2020;20(5):3602‐3610. 32272018 10.1021/acs.nanolett.0c00529

[19] Shi S , Fu W , Lin S , et al. Targeted and effective glioblastoma therapy via aptamer‐modified tetrahedral framework nucleic acid‐paclitaxel Nanoconjugates that can pass the blood brain barrier. Nanomed Nanotechnol Biol Med. 2019;21:102061. 10.1016/j.nano.2019.102061 31344499

[20] Zhao D , Xiao D , Liu M , et al. Tetrahedral framework nucleic acid carrying angiogenic peptide prevents bisphosphonate‐related osteonecrosis of the jaw by promoting angiogenesis. Int J Oral Sci. 2022;14(1):23. 35477924 10.1038/s41368-022-00171-7 PMC9046247

[21] Zhang Q , Lin S , Wang L , et al. Tetrahedral framework nucleic acids act as antioxidants in acute kidney injury treatment. Chem Eng J. 2021;413:127426.

[22] Zhou M , Gao S , Zhang X , et al. The protective effect of tetrahedral framework nucleic acids on periodontium under inflammatory conditions. Bioactive Mater. 2021;6(6):1676‐1688. 10.1016/j.bioactmat.2020.11.018 PMC7708773 33313447

[23] Lin S , Zhang Q , Li S , et al. Antioxidative and angiogenesis‐promoting effects of tetrahedral framework nucleic acids in diabetic wound healing with activation of the Akt/Nrf2/HO‐1 pathway. ACS Appl Mater Interfaces. 2020;12:11397‐11408. 32083455 10.1021/acsami.0c00874

[24] Love MI , Huber W , Anders S . Moderated estimation of fold change and dispersion for RNA‐seq data with DESeq2. Genome Biol. 2014;15(12):550. 25516281 10.1186/s13059-014-0550-8 PMC4302049

[25] Wu T , Hu E , Xu S , Chen M , Yu G . clusterProfiler 4.0: a universal enrichment tool for interpreting omics data. Innovation. 2021;2(3):5526‐5542. 10.1016/j.xinn.2021.100141 PMC8454663 34557778

[26] Zhou X , Lai Y , Xu X , et al. Tetrahedral framework nucleic acids inhibit pathological neovascularization and vaso‐obliteration in ischaemic retinopathy via PI3K/AKT/mTOR signalling pathway. Cell Prolif. 2023;56(7):e13407. 36694349 10.1111/cpr.13407 PMC10334269

[27] Li J , Xiao L , Yan N , et al. The neuroprotective effect of MicroRNA‐22‐3p modified tetrahedral framework nucleic acids on damaged retinal neurons via TrkB/BDNF signaling pathway. Adv Funct Mater. 2021;31(36):2104141.

[28] Shi S , Tian T , Li Y , et al. Tetrahedral framework nucleic acid inhibits chondrocyte apoptosis and oxidative stress through activation of autophagy. ACS Appl Mater Interfaces. 2020;12(51):56782‐56791. 33289541 10.1021/acsami.0c17307

[29] Hu P‐F , Chen W‐P , Bao J‐P , Wu L‐D . Paeoniflorin inhibits IL‐1β‐induced chondrocyte apoptosis by regulating the Bax/Bcl‐2/caspase‐3 signaling pathway. Mol Med Rep. 2018;17(4):6194‐6200. 29484390 10.3892/mmr.2018.8631

[30] Oh SE , Mouradian MM . Cytoprotective mechanisms of DJ‐1 against oxidative stress through modulating ERK1/2 and ASK1 signal transduction. Redox Biol. 2018;14:211‐217. 28954246 10.1016/j.redox.2017.09.008 PMC5614756

[31] Kundu S , Saadi F , Sengupta S , et al. DJ‐1‐Nrf2 axis is activated upon murine β‐coronavirus infection in the CNS. Brain Disorders. 2021;4:100021. 34514445 10.1016/j.dscb.2021.100021 PMC8418700

[32] Stitt AW , Curtis TM , Chen M , et al. The progress in understanding and treatment of diabetic retinopathy. Prog Retin Eye Res. 2016;51:156‐186. 26297071 10.1016/j.preteyeres.2015.08.001

[33] Aragonès G , Rowan S , Francisco G , et al. Glyoxalase system as a therapeutic target against diabetic retinopathy. Antioxidants. 2020;9(11):1062. 33143048 10.3390/antiox9111062 PMC7692619

[34] Dash BK , Urano Y , Saito Y , Noguchi N . Redox‐sensitive DJ‐1 protein: an insight into physiological roles, secretion, and therapeutic target. Redox Exper Med. 2022;2022(1):R96‐R115.

[35] Nashine S . Potential therapeutic candidates for age‐related macular degeneration (AMD). Cells. 2021;10(9):2483. 34572131 10.3390/cells10092483 PMC8464988

[36] Ibba ML , Ciccone G , Esposito CL , Catuogno S , Giangrande PH . Advances in mRNA non‐viral delivery approaches. Adv Drug Deliv Rev. 2021;177:113930. 34403751 10.1016/j.addr.2021.113930

[37] Wang J , Place RF , Portnoy V , et al. Inducing gene expression by targeting promoter sequences using small activating RNAs. J Biol Methods. 2015;2(1):e14. 25839046 10.14440/jbm.2015.39 PMC4379447

