# Supplementary material for: The antioxidant effect of tetrahedral framework nucleic acid‐based delivery of small activating RNA targeting DJ‐1 on retinal oxidative stress injury
Source: Cell Prolif. 2024 Apr 9;57(8):e13635. doi: 10.1111/cpr.13635 (PMC11294416; doi:10.1111/cpr.13635)
Supplement: Supplementary file 1 — Data S1. Supporting Information. [file CPR-57-e13635-s003.pdf]

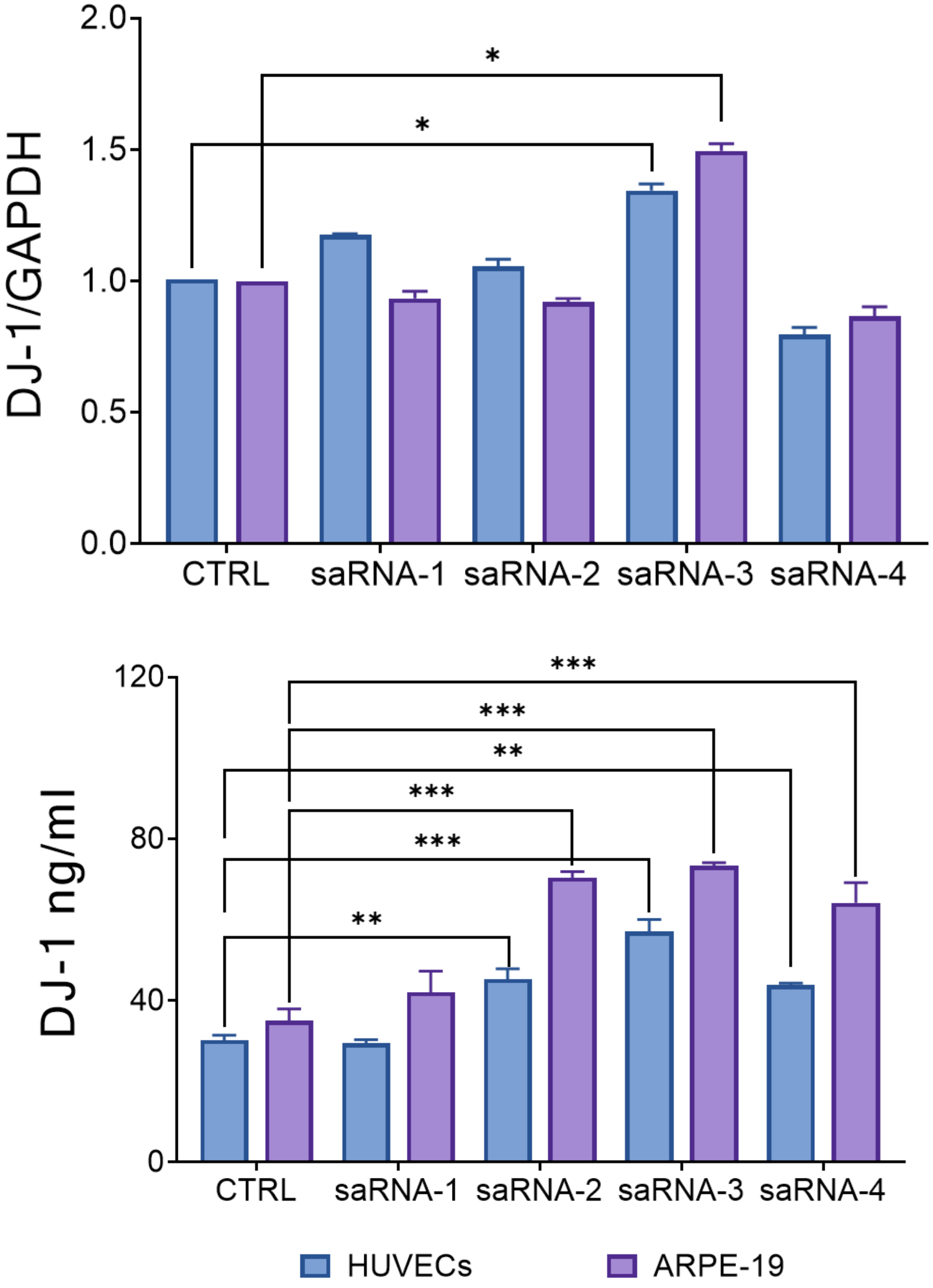

Figure S1. The expression of DJ-1 in HUVECs and ARPE-19 cells was detected by real-time quantitative PCR and ELISA

A, Real-time quantitative PCR (RT-qPCR) was used to measure the expression of DJ-1 mRNA in HUVECs and ARPE-19 cells at 24 hours after transfection with 4 different saRNA. GAPDH was used as an internal control. B, ELISA was used to quantify the expression of DJ-1 protein in HUVECs and ARPE-19 cells at 24 hours after transfection with 4 different saRNA, respectively. Statistical analysis: ns $p \geq 0.05$, * $p < 0.05$, ** $p < 0.01$, *** $p < 0.001$.

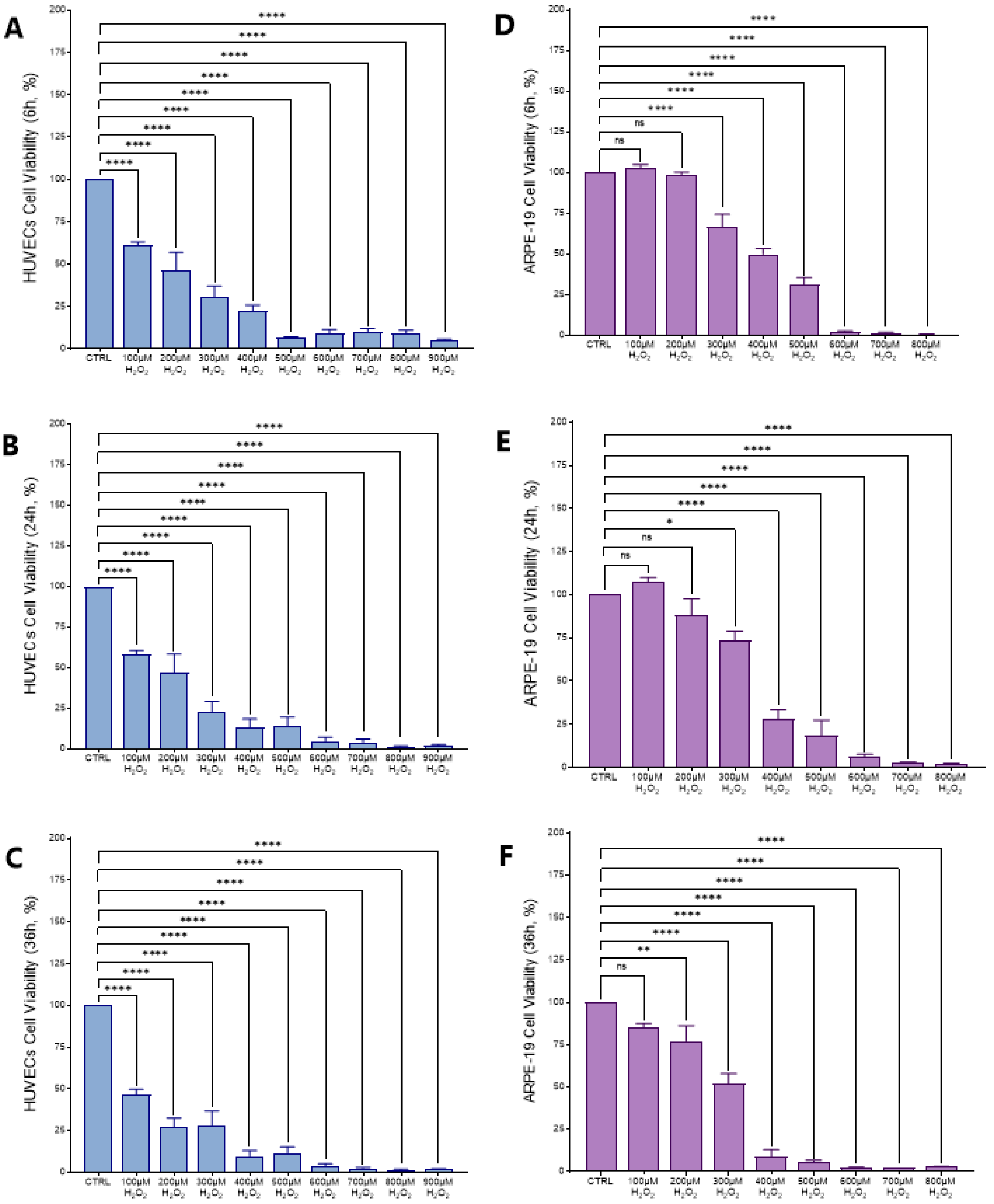

Figure S2. Cell viability of HUVECs and ARPE-19 cells treated in different concentrations of H_2O_2 for 6h, 24h, and 36hand Gestation Age of All Included Infants

After cultured in blank medium containing 100-900μM H_2O_2 for 6h (A), 24h (B), and 36h (C), the cell viability of HUVECs was detected by CCK-8. The cell viability of HUVECs treated with different concentrations of H_2O_2 showed a concentration-dependent manner. Compared with the control group (CTRL), the cell viability of HUVECs was significantly decreased after 100-900μM H_2O_2 treatment (all $p < 0.0001$). Then, CCK-8 was used to detect the cell viability of ARPE-19 cells after cultured in blank medium containing 100-800μM H_2O_2 for 6h (D), 24h (E), and 36h (F). The cell viability of ARPE-19 cells increased after 6 and 24 hours of treatment with 100μM H_2O_2, but the difference was not statistically significant (all $p \geq 0.05$). The cell viability of ARPE-19 cells decreased after 6 and 24 hours of treatment with 200μM H_2O_2 and 36 hours of treatment with 100μM H_2O_2, and the difference was not statistically significant (all $p \geq 0.05$). Compared with the CTRL group, the cell viability of ARPE-19 cells treated with 300-800μM H_2O_2 for 6 and 24 hours and with 200-800μM H_2O_2 for 36 hours decreased significantly (all $p < 0.05$). Statistical analysis: ns $p \geq 0.05$, * $p < 0.05$, ** $p < 0.01$, **** $p < 0.0001$. Data are presented as mean ± SD (n = 3).

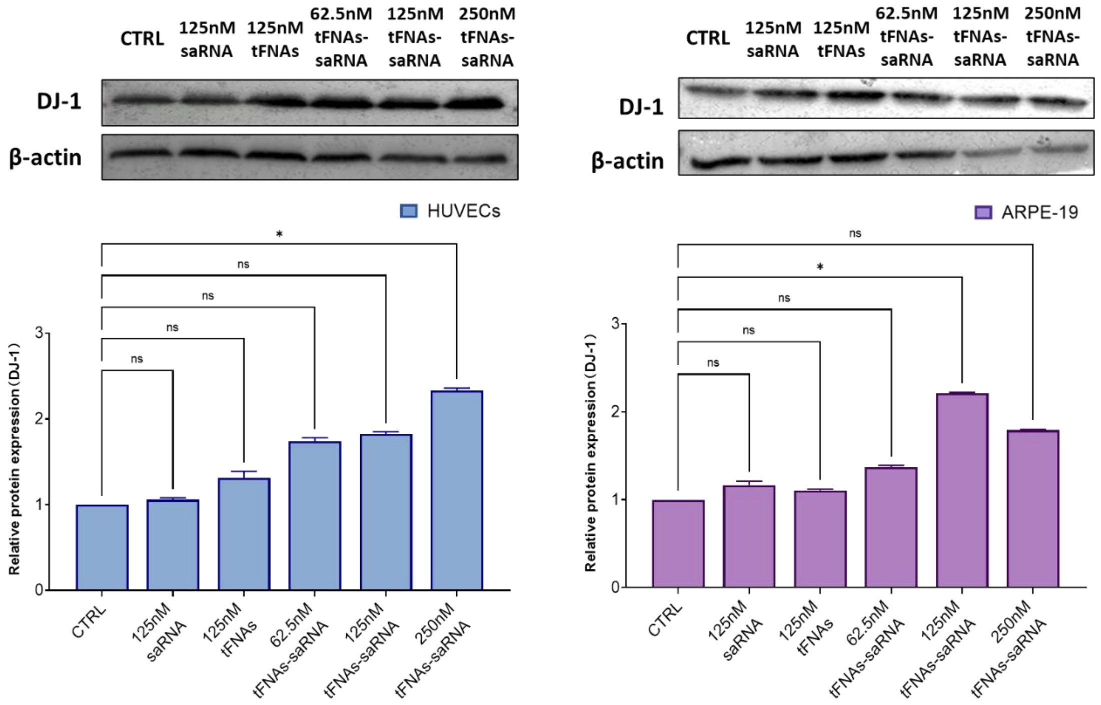

Figure S3. Western blotting showing the level of DJ-1 protein in HUVEC and ARPE-19 after treatment of different concentrations of tFNAs-DJ-1-saRNA

Data are presented as mean ± SD (n = 3). Statistical analysis: ns, no significance, $p \geq 0.05$, * $p < 0.05$, ** $p < 0.01$, **** $p < 0.0001$.

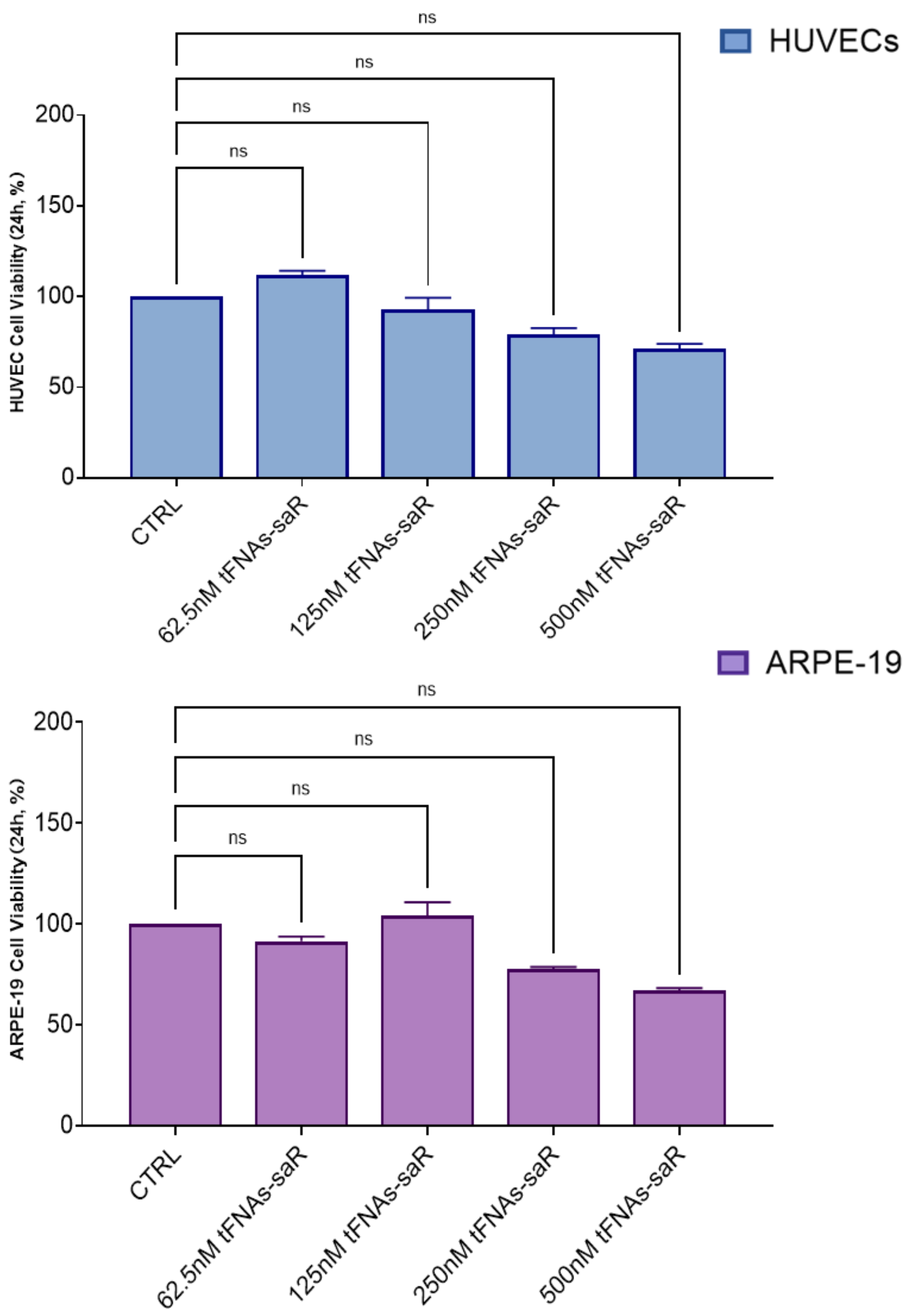

Figure S4. Cell viability of HUVECs and ARPE-19 cells treated with different concentrations of tFNAs-DJ-1-saRNA for 24h

Data are presented as mean ± SD ($n = 3$). Statistical analysis: ns, no significance, $p \geq 0.05$, * $p < 0.05$, ** $p < 0.01$, **** $p < 0.0001$.

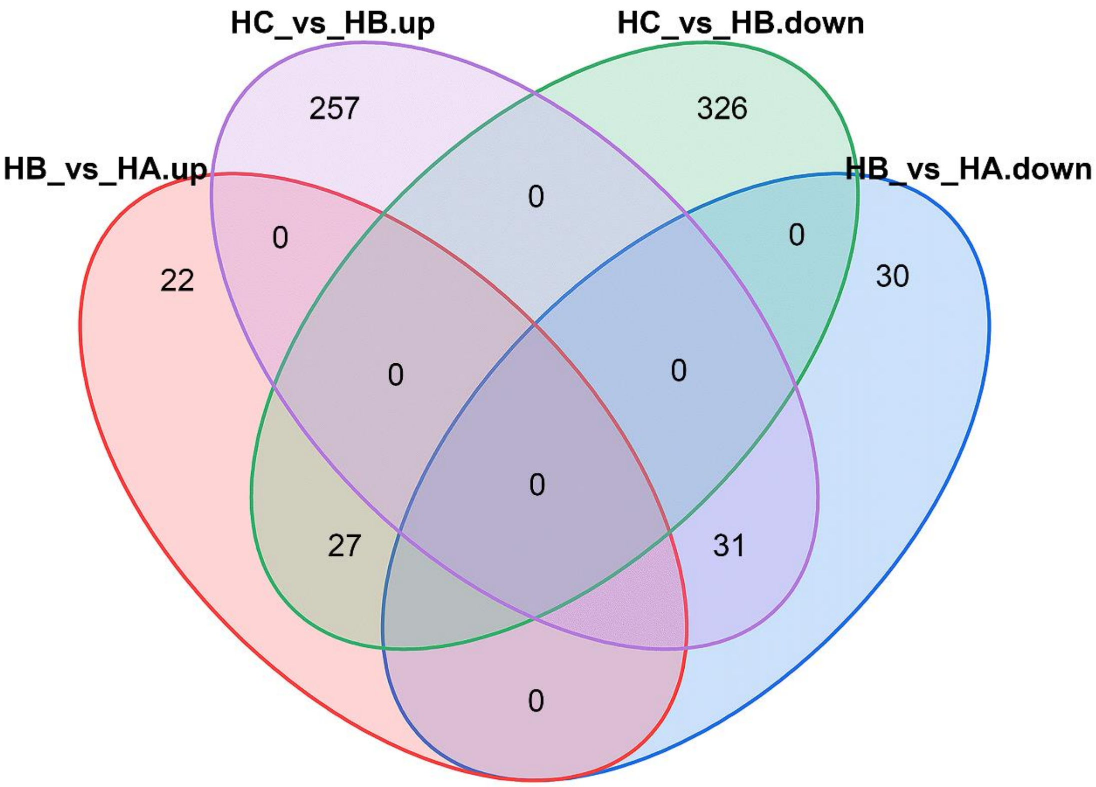

Figure S5A. The Venn plot showed that the expression of DEGs between H2O2 treatment(HA) and the control(HC) group, tFNAs-DJ-1-saRNA(HB) and H2O2 treatment (HA) group in HUVEC.

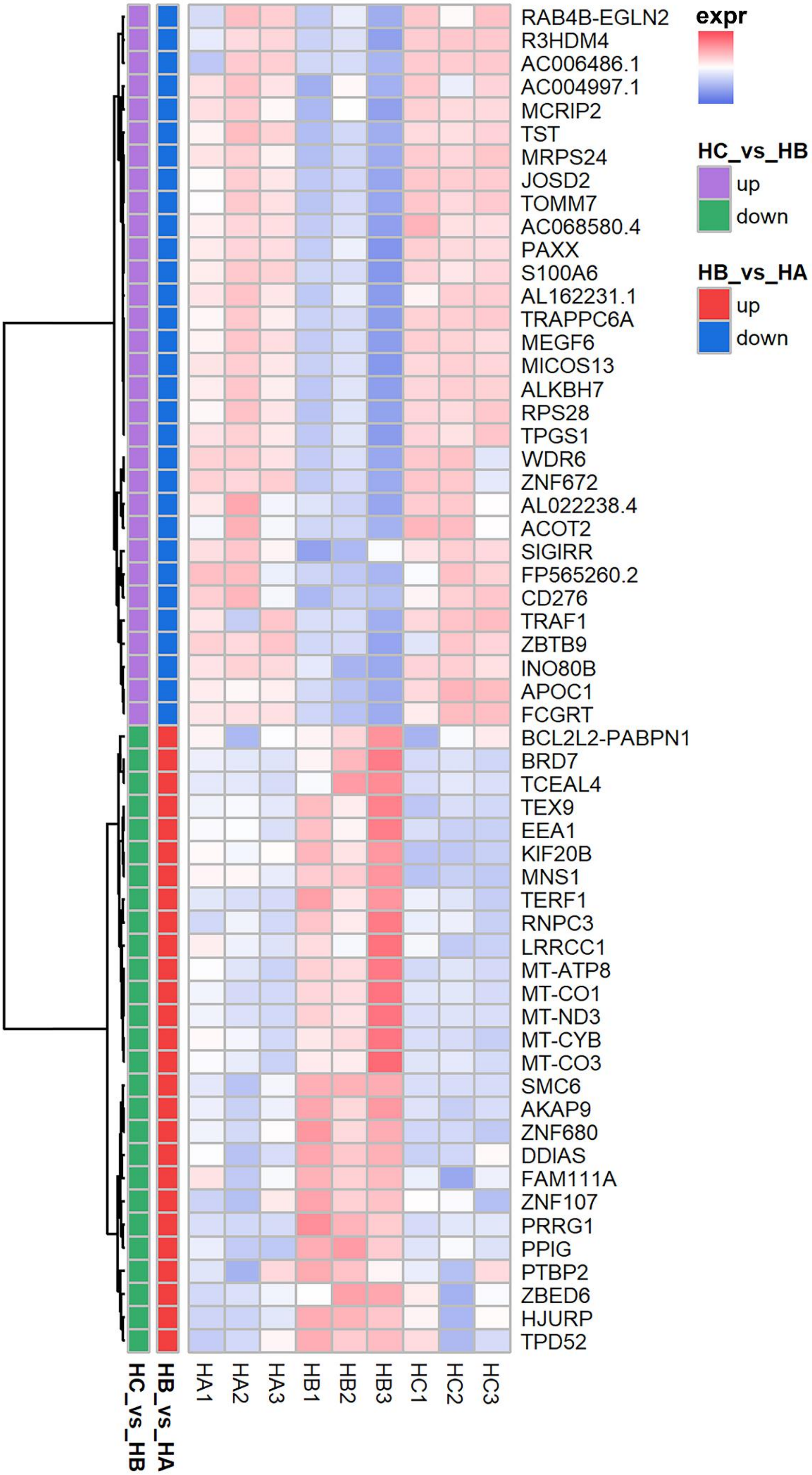

Figure S5B. The heatmap showed that the expression of DEGs between H2O2 treatment(HA) and the control(HC) group, tFNAs-DJ-1-saRNA(HB) and H2O2 treatment (HA) group in HUVEC.
