# Supplementary material for: The antioxidant effect of tetrahedral framework nucleic acid‐based delivery of small activating RNA targeting DJ‐1 on retinal oxidative stress injury
Source: Cell Prolif. 2024 Apr 9;57(8):e13635. doi: 10.1111/cpr.13635 (PMC11294416; doi:10.1111/cpr.13635)
Supplement: Supplementary file 2 — Table S1. Base sequence of each ssDNA and saRNA. [file CPR-57-e13635-s001.docx]

Table S1 Base sequence of each ssDNA and saRNA

| ssDNA | Base sequence (from 5’ to 3’) |
| --- | --- |
| S1 | TTGACCTGTGAATTATTTATCACCCGCCATAGTAGACGTATCACCAGGCAGTTGAGACGAACATTCCTAAGTCTGAA |
| S1-saRNA-guide strand | ATTTATCACCCGCCATAGTAGACGTATCACCAGGCAGTTGAGACGAACATTAATAAGTCTGAATTTTTCAGGGCUGUCCAGCUAGAA |
| saRNA-guide strand | UUCACAGGUCAACAGGGCUGUCCAGCUAGAA |
| saRNA-passenger strand | UUCUAGCUGGACAGCCCUG |
| S2 | ACATGCGAGGGTCCAATACCGACGATTACAGCTTGCTACACGATTCAGACTTAGGAATGTTCG |
| S3 | ACTACTATGGCGGGTGATAAAACGTGTAGCAAGCTGTAATCGACGGGAAGAGCATGCCCATCC |
| S4 | ACGGTATTGGACCCTCGCATGACTCAACTGCCTGGTGATACGAGGATGGGCATGCTCTTCCCG |
