# Supplementary material for: The antioxidant effect of tetrahedral framework nucleic acid‐based delivery of small activating RNA targeting DJ‐1 on retinal oxidative stress injury
Source: Cell Prolif. 2024 Apr 9;57(8):e13635. doi: 10.1111/cpr.13635 (PMC11294416; doi:10.1111/cpr.13635)
Supplement: Supplementary file 3 — Table S2. The sequences of PCR primers. [file CPR-57-e13635-s002.docx]

**Table S2 The sequences of PCR primers**

| Gene | Primer sequence (5’-3’) |
| --- | --- |
| DJ-1 | 5’-GCTCTGTTGGCTCATGAAATAG-3’ |
|  | 5’-CAGAGTAGGTGTAATGACCTCC-3’ |
| Bax | 5’-TTGCCCTCTTCTACTITGCTAG-3’ |
|  | 5’-CCATGATGGTTCTGATCAGCTC-3’ |
| Bcl-2 | 5’-GATGACTTCTCTCGTCGCTAC-3’ |
|  | 5’-GAACTCAAAGAAGGCCACAATC-3’ |
| Caspase-3 | 5’-GAAACTCTTCATCATTCAGGCC-3’ |
|  | 5’-GCGAGTGAGAATGTGCATAAT-3’ |
| GAPDH | 5’-ACCACAGTCCATGCCATCAC-3’ |
|  | 5’-TCCACCACCCTGTTGCTGTA-3’ |
